# Protecting against Pneumococcal Disease: Critical Interactions between Probiotics and the Airway Microbiome

**DOI:** 10.1371/journal.ppat.1002652

**Published:** 2012-06-07

**Authors:** Paul V. Licciardi, Zheng Quan Toh, Eileen Dunne, Sook-San Wong, Edward K. Mulholland, Mimi Tang, Roy M. Robins-Browne, Catherine Satzke

**Affiliations:** 1 Pneumococcal Research, Murdoch Childrens Research Institute, Royal Children's Hospital, Melbourne, Australia; 2 Allergy and Immune Disorders, Murdoch Childrens Research Institute, Royal Children's Hospital, Melbourne, Australia; 3 London School of Hygiene and Tropical Medicine, London, United Kingdom; 4 Menzies School of Health Research, Darwin, Australia; 5 Allergy and Immunology, Royal Children's Hospital, Melbourne, Australia; 6 Department of Paediatrics, The University of Melbourne, Melbourne, Australia; 7 Department of Microbiology and Immunology, The University of Melbourne, Melbourne, Australia; 8 Infectious Diseases and Microbiology, Murdoch Childrens Research Institute, Royal Children's Hospital, Melbourne, Australia; The Fox Chase Cancer Center, United States of America

*Streptococcus pneumoniae* (the pneumococcus) is a predominant cause of pneumonia, meningitis, and bacteremia. It is a leading killer of children under 5 years of age, responsible for the deaths of up to 2 million children annually [1]. Most deaths occur in African and Asian developing countries; however, pneumococcal disease is also a significant problem in particular populations of developed countries, such as the North American Indians, and indigenous Alaskans and Australians [1]–[3]. Although vaccination is the most cost-effective method of protection against pneumococcal disease, cost remains a barrier, as does vaccine delivery and efficacy. In this opinion piece, we discuss the potential complementary role of probiotics to vaccines in preventing pneumococcal disease through targeting the microbiome of the upper respiratory tract.

A prerequisite for pneumococcal disease is adherence of the bacterium to host nasopharyngeal epithelium leading to colonization (carriage). The mucosal surface and the microbiome of the nasopharynx are thought to protect against carriage [4]. Vaccination with pneumococcal vaccines reduces carriage of the organism, and the risk of invasive disease caused by vaccine serotypes and some cross-reactive non-vaccine serotypes. Moreover, vaccines generate herd immunity that may protect unvaccinated individuals against infection [5].

In North America and other developed regions, >80% of pediatric invasive pneumococcal disease (IPD) is accounted for by serotypes contained within the first-generation seven serotype conjugate vaccine (PCV7, Prevnar, Wyeth/Pfizer, United States). In high-risk populations, several factors diminish the efficacy of pneumococcal vaccines. For example, PCV7 protects against only ∼50% of serotypes causing IPD in developing countries of Africa and Asia [6]. Pneumococcal conjugate vaccines are also too expensive for resource-poor countries that experience the overwhelming burden of disease globally. The GAVI Alliance has made significant inroads to this problem, providing access to these and other life-saving vaccines to children most in need at a cost of US$1 billion per year [7]. Nevertheless, complete vaccine delivery is another major public health challenge. While GAVI is planning to implement pneumococcal conjugate vaccines in 19 developing countries over the next 2 years [8], vaccine uptake may be more difficult in certain populations. Amongst indigenous Australians, <50% of infants aged 7 months have received the full three-dose schedule (at 2, 4, and 6 months) [9], providing suboptimal protection against colonization and disease. In many countries, the first PCV7 dose is received after colonization has occurred—usually within the first 6 weeks of life—which may further limit the efficacy of pneumococcal vaccination.

Furthermore, serotype replacement is considered the most significant problem in the post-PCV7 era. Elimination of vaccine-serotype carriage has provided new niches for colonization and subsequent rises in invasive disease with non-PCV7 serotypes [10]. Although licensure of higher valency PCVs containing ten or 13 serotypes would be expected to reduce serotype replacement, the emergence of other invasive serotypes is likely.

Other early life strategies to prevent pneumococcal disease are needed, particularly for resource-poor settings. Maternal and neonatal immunization approaches are currently under investigation for their impact on disease during the first weeks of life. Targeting the microbiome to modulate colonization has been postulated as one mechanism to improve the efficacy of a range of vaccines against multiple pathogens [11]. It has now been demonstrated that in early infancy, colonization with pneumococci prior to conjugate vaccination causes impaired immune responses to the carried serotype [12], [13]. Exploiting the beneficial effects of probiotics on microbial colonization and immunity represents a novel approach to prevent or reduce pneumococcal colonization and disease.

The World Health Organization (WHO) defines probiotics as live micro-organisms that confer a health benefit to the host and are generally regarded as safe in humans [14]. Moreover, clinical studies have confirmed the safety and feasibility of oral administration of probiotics in infancy [15], [16]. *Lactobacillus* and *Bifidobacterium* are the two most widely studied genera of probiotic bacteria [17]. Probiotic activity is highly species- and strain-specific [18], [19]. Principal amongst their pleiotropic effects is the capacity to counteract microbiome disturbances, suggesting the potential to modulate pneumococcal colonization [20]. Indeed, experimental data suggest that probiotics can influence the profile of microbial species in the nasopharynx to reduce pneumococcal colonization [21]–[24]. Probiotics also maintain epithelial barrier integrity and modulate systemic and mucosal immune responses [14]. Furthermore, probiotic-microbiome crosstalk is important, as intestinal microbiota can shape immune responses by controlling the relative activity of regulatory T cells and Th17 cells [25], [26]. A paradigm for the effects of probiotics in modulating host responses in the nasopharynx to protect against pneumococcal infection is proposed in Figure 1. Importantly, while the mechanisms of action proposed are largely supported by animal studies, more research is needed to confirm these effects in humans.

**Figure 1:**
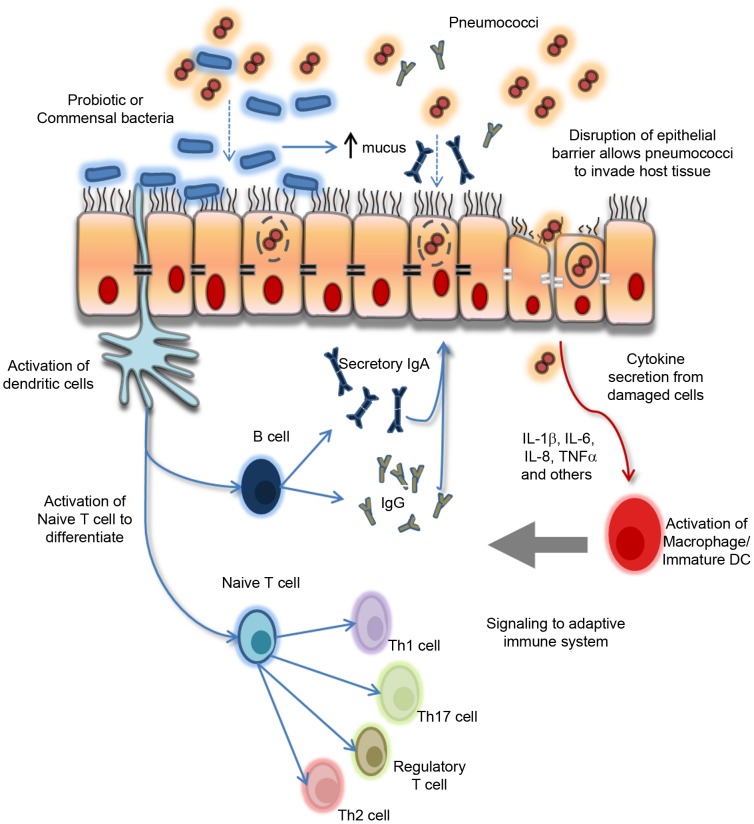
Paradigm for the proposed biological effects of probiotic bacteria in protection against pneumococcal infection. Commensal and/or probiotic bacteria can prevent pathogens (pneumococci) from attaching to and colonizing the respiratory epithelium by associating with specific cell surface receptors and by enhancing mucus secretion and the production of secretory IgA. Probiotic bacteria interact with underlying dendritic cells (DCs) which signal to the adaptive immune system to trigger a variety of effector cell types, including Th1, Th2, and Th17 as well as regulatory T cells and B cells depending on the local cytokine/chemokine microenvironment. Furthermore, probiotic bacteria also maintain the epithelial barrier integrity by upregulating the expression of specific tight junction proteins on damaged epithelium as a result of localized inflammatory responses following pathogen (pneumococcal) encounter and invasion. Refer to references [49]–[52] for more detail on probiotic–host effects. Th, T helper cell.

Probiotics show specificity in their effect on microbial patterns in the nasopharynx. Most of the available data is based on animal models of colonization or disease. For example, in a mouse model of pneumococcal pneumonia, *Lactobacillus lactis* lowered lung colonization and increased specific IgG and IgA levels in bronchoalveolar secretions after challenge with pneumococcus serotype 14 [21], while *Lactobacillus fermentum* reduced nasopharyngeal colonization after challenge with pneumococcal serotype 6A [22]. In humans, the potential for probiotics to have an impact on airway microbial colonization is less clear. In 108 adult volunteers given a probiotic yogurt containing *Lactobacillus rhamnosus* GG (LGG), *Bifidobacterium* sp. B420, *Lactobacillus acidophilus* 145, and *Streptococcus thermophilus*, a significant reduction in pathogenic bacteria (including *Staphylococcus aureus*, *S. pneumoniae*, beta-hemolytic streptococci, and *Haemophilus influenzae*) was observed compared to a standard yogurt [24]. *Streptococcus salivarius* is suggested to be an appropriate probiotic species given that it is a known colonizer of the upper respiratory tract in humans [27]. It has been shown to produce bacteriocin-like substances with inhibitory activities against a number of important airway pathogens in vitro and in vivo [27], [28] as well as possess immunomodulatory properties in vitro [29], [30]. In otitis media-prone children given antibiotics prior to oral treatment with a powdered *S. salivarius* K12 formula, 33% were newly colonized with K12 while two of 19 children were shown to expand the pre-existing *S. salivarius* population [31]. No impact on clinical outcomes was reported in this study, and the small sample size used makes it difficult to draw meaningful conclusions. In contrast, when otitis-prone children (*n* = 155) were given a daily probiotic mix containing LGG, *L. rhamnosus* LC705, *B. breve* 99, and *Propionibacterium freudenreichii* JS for 24 weeks, no effect on nasopharyngeal carriage of otitis pathogens was observed. Furthermore, this probiotic formula did not prevent the occurrence of otitis media in these children, although there was a trend of reduced recurrent respiratory infections [32]. Taken together, the evidence of probiotic effects in human studies is more limited compared to animal models and justifies the continued investigation of candidate probiotic species such as *S. salivarius* and lactobacilli on airway microbial colonization and their mechanisms of action.

To date, the effect of probiotics on the gastrointestinal microbiome have provided the best evidence for host–microbe interactions such as pathogen exclusion, enhanced mucus secretion, production of anti-bacterial factors, and modulation of host immunity [14]. Probiotics can restore aberrant microbiota patterns associated with inflammatory diseases such as Crohn's Disease [33] and allergy [17]. Several clinical studies have shown that infants who later develop atopic dermatitis have altered microbiota, with greater numbers of pathogenic clostridial and staphylococcal species and fewer beneficial bifidobacteria [34], [35]. Importantly, dysbiosis precedes clinical symptoms of allergy [36], indicating a causal relationship between altered microbiota and disease. Administration of LGG modulates the composition of the intestinal microbiota in allergic infants, and reduced by half the incidence of atopic dermatitis in high-risk infants by age 2 [36], [37]. LGG also corrected dysbiosis and reduced disease severity in a mouse model of colitis [38].

These data have implications for pneumococcal disease. Importantly, lung immunity is affected by the intestinal microbiome, which induces Th1 and IgA responses via specific inflammasomes [39]. Therefore, modulation of inflammasome activity by probiotics represents a key biological target. The balance between microbiome status and health are also linked to the production of potent anti-inflammatory short-chain fatty acids such as butyrate and acetate [40]. Probiotics restore short-chain fatty acid levels, and the protective effects of *Bifidobacteria* species against enterohemorrhagic *E. coli* infection was shown to be dependent on acetate production [41].

Probiotics also appear to play an important role in facilitating mucosal immunity against infection [42]. Specifically, probiotics are demonstrated to be effective vaccine adjuvants, enhancing IgG- and IgA-specific responses to parenteral and mucosal vaccines such as influenza [43], *H. influenzae* type b (Hib) [44], polio [45], rotavirus [46], and *Salmonella typhi* [47] in humans. More studies on the adjuvant properties of probiotics in humans are needed, as the effects reported are often variable and have been based on clinical trials involving small sample sizes. For example, in the study by Fang et al. [47], treatment with LGG or *L. lactis* did not significantly enhance the IgG or IgA response to an oral *S. typhi* Ty21a vaccine despite LGG increasing *S. typhi*–specific IgA antibody secreting cells in a greater number of subjects than *L. lactis* or placebo. Similarly, while supplementation with a *Bifidobacterium longum* BL999 and *L. rhamnosus* LPR mix to infants doubled the anti-HBsAg IgG levels following vaccination compared to placebo, this was not statistically significant [48]. In a study by Kukkonen et al. [44], daily administration of a LGG, *L. rhamnosus* LC705, *B. breve* Bbi99, and *Propionibacterium freudenreichii* combination to mothers in the last 4 weeks of pregnancy, and to their infants for the first 6 months of life, increased the Hib-specific IgG response in infants. However, no change in diphtheria toxoid or tetanus toxoid IgG levels was observed, suggesting that the effects of probiotics may vary depending on the vaccine antigen used. Recently, *Lactobacillus casei* was reported to significantly enhance the pneumococcal protective protein A (PppA)-specific IgG and IgA response in the serum and mucosa following nasal vaccination with PppA and was associated with a significantly reduced pathogen load in the nasal lavage by day 42 post-immunization [42]. Despite this, the adjuvant activity of probiotics following pneumococcal vaccination in humans is unknown and remains an intriguing prospect for further research.

The promising findings of these studies has made it increasingly clear that significant research emphasis on reducing pneumococcal colonization during the neonatal period is warranted, ideally involving human clinical trials. Novel early life strategies that reduce infection with *S. pneumoniae* may have important health benefits, especially in high-risk populations. The combined effects of modulating the nasopharyngeal microbiome and enhanced mucosal immunity justify the continued investigation of probiotics for protection against pneumococcal infection.

## References

[1] O'Brien KL, Wolfson LJ, Watt JP, Henkle E, Deloria-Knoll M (2009). Burden of disease caused by *Streptococcus pneumoniae* in children younger than 5 years: global estimates.. Lancet.

[2] Said MA, O'Brien KL, Nuorti JP, Singleton R, Whitney CG (2011). The epidemiologic evidence underlying recommendations for use of pneumococcal polysaccharide vaccine among American Indian and Alaska Native populations.. Vaccine.

[3] Jacups SP, Cheng A (2011). The epidemiology of community acquired bacteremic pneumonia, due to *Streptococcus pneumoniae*, in the Top End of the Northern Territory, Australia–over 22 years.. Vaccine.

[4] Malley R (2010). Antibody and cell-mediated immunity to *Streptococcus pneumoniae*: implications for vaccine development.. J Mol Med (Berl).

[5] Isaacman DJ, Strutton DR, Kalpas EA, Horowicz-Mehler N, Stern LS (2008). The impact of indirect (herd) protection on the cost-effectiveness of pneumococcal conjugate vaccine.. Clin Ther.

[6] Johnson HL, Deloria-Knoll M, Levine OS, Stoszek SK, Freimanis Hance L (2010). Systematic evaluation of serotypes causing invasive pneumococcal disease among children under five: the pneumococcal global serotype project.. PLoS Med.

[7] Lob-Levyt J (2011). Contribution of the GAVI Alliance to improving health and reducing poverty.. Philos Trans R Soc Lond B Biol Sci.

[8] Nossal GJ (2011). Vaccines and future global health needs.. Philos Trans R Soc Lond B Biol Sci.

[9] O'Grady KA, Krause V, Andrews R (2009). Immunisation coverage in Australian Indigenous children: time to move the goal posts.. Vaccine.

[10] Melegaro A, Choi YH, George R, Edmunds WJ, Miller E (2010). Dynamic models of pneumococcal carriage and the impact of the Heptavalent Pneumococcal Conjugate Vaccine on invasive pneumococcal disease.. BMC Infect Dis.

[11] Ferreira RB, Antunes LC, Finlay BB (2010). Should the human microbiome be considered when developing vaccines?. PLoS Pathog.

[12] Dagan R, Givon-Lavi N, Greenberg D, Fritzell B, Siegrist CA (2010). Nasopharyngeal carriage of *Streptococcus pneumoniae* shortly before vaccination with a pneumococcal conjugate vaccine causes serotype-specific hyporesponsiveness in early infancy.. J Infect Dis.

[13] Rodenburg GD, van Gils EJ, Veenhoven RH, Bogaert D, van den Dobbelsteen GP (2011). Lower immunoglobulin G antibody responses to pneumococcal conjugate vaccination at the age of 2 years after previous nasopharyngeal carriage of *Streptococcus pneumoniae*.. J Pediatr.

[14] Gareau MG, Sherman PM, Walker WA (2010). Probiotics and the gut microbiota in intestinal health and disease.. Nat Rev Gastroenterol Hepatol.

[15] Braegger C, Chmielewska A, Decsi T, Kolacek S, Mihatsch W (2011). Supplementation of infant formula with probiotics and/or prebiotics: a systematic review and comment by the ESPGHAN committee on nutrition.. J Pediatr Gastroenterol Nutr.

[16] Boyle RJ, Robins-Browne RM, Tang ML (2006). Probiotic use in clinical practice: what are the risks?. Am J Clin Nutr.

[17] Tang ML, Lahtinen SJ, Boyle RJ (2010). Probiotics and prebiotics: clinical effects in allergic disease.. Curr Opin Pediatr.

[18] Bron PA, van Baarlen P, Kleerebezem M (2011). Emerging molecular insights into the interaction between probiotics and the host intestinal mucosa.. Nat Rev Microbiol.

[19] Yan F, Polk DB (2011). Probiotics and immune health.. Curr Opin Gastroenterol.

[20] Reid G, Younes JA, Van der Mei HC, Gloor GB, Knight R (2011). Microbiota restoration: natural and supplemented recovery of human microbial communities.. Nat Rev Microbiol.

[21] Medina M, Villena J, Salva S, Vintini E, Langella P (2008). Nasal administration of *Lactococcus lactis* improves local and systemic immune responses against *Streptococcus pneumoniae*.. Microbiol Immunol.

[22] Cangemi de Gutierrez R, Santos V, Nader-Macias ME (2001). Protective effect of intranasally inoculated *Lactobacillus fermentum* against *Streptococcus pneumoniae* challenge on the mouse respiratory tract.. FEMS Immunol Med Microbiol.

[23] Racedo S, Villena J, Medina M, Aguero G, Rodriguez V (2006). *Lactobacillus casei* administration reduces lung injuries in a *Streptococcus pneumoniae* infection in mice.. Microbes Infect.

[24] Gluck U, Gebbers JO (2003). Ingested probiotics reduce nasal colonization with pathogenic bacteria (*Staphylococcus aureus, Streptococcus pneumoniae*, and beta-hemolytic streptococci).. Am J Clin Nutr.

[25] Niess JH, Leithauser F, Adler G, Reimann J (2008). Commensal gut flora drives the expansion of proinflammatory CD4 T cells in the colonic lamina propria under normal and inflammatory conditions.. J Immunol.

[26] O'Mahony C, Scully P, O'Mahony D, Murphy S, O'Brien F (2008). Commensal-induced regulatory T cells mediate protection against pathogen-stimulated NF-kappaB activation.. PLoS Pathog.

[27] Walls T, Power D, Tagg J (2003). Bacteriocin-like inhibitory substance (BLIS) production by the normal flora of the nasopharynx: potential to protect against otitis media?. J Med Microbiol.

[28] Santagati M, Scillato M, Patanè F, Aiello C, Stefani S (2012). Bacteriocin-producing oral streptococci and inhibition of respiratory pathogens.. FEMS Immunol Med Microbiol.

[29] Guglielmetti S, Taverniti V, Minuzzo M, Arioli S, Stuknyte M (2010). Oral bacteria as potential probiotics for the pharyngeal mucosa.. Appl Environ Microbiol.

[30] Cosseau C, Devine DA, Dullaghan E, Gardy JL, Chikatamarla A (2008). The commensal *Streptococcus salivarius* K12 downregulates the innate immune responses of human epithelial cells and promotes host-microbe homeostasis.. Infect Immun.

[31] Power DA, Burton JP, Chilcott CN, Dawes PJ, Tagg JR (2008). Preliminary investigations of the colonisation of upper respiratory tract tissues of infants using a paediatric formulation of the oral probiotic *Streptococcus salivarius* K12.. Eur J Clin Microbiol Infect Dis.

[32] Hatakka K, Blomgren K, Pohjavuori S, Kaijalainen T, Poussa T (2007). Treatment of acute otitis media with probiotics in otitis-prone children-a double-blind, placebo-controlled randomised study.. Clin Nutr.

[33] Thomas DW, Greer FR (2010). Probiotics and prebiotics in pediatrics.. Pediatrics.

[34] Kalliomaki M, Kirjavainen P, Eerola E, Kero P, Salminen S (2001). Distinct patterns of neonatal gut microflora in infants in whom atopy was and was not developing.. J Allergy Clin Immunol.

[35] Watanabe S, Narisawa Y, Arase S, Okamatsu H, Ikenaga T (2003). Differences in fecal microflora between patients with atopic dermatitis and healthy control subjects.. J Allergy Clin Immunol.

[36] Kalliomaki M, Salminen S, Arvilommi H, Kero P, Koskinen P (2001). Probiotics in primary prevention of atopic disease: a randomised placebo-controlled trial.. Lancet.

[37] Lahtinen SJ, Boyle RJ, Kivivuori S, Oppedisano F, Smith KR (2009). Prenatal probiotic administration can influence *Bifidobacterium* microbiota development in infants at high risk of allergy.. J Allergy Clin Immunol.

[38] Sokol H, Pigneur B, Watterlot L, Lakhdari O, Bermudez-Humaran LG (2008). *Faecalibacterium prausnitzii* is an anti-inflammatory commensal bacterium identified by gut microbiota analysis of Crohn disease patients.. Proc Natl Acad Sci U S A.

[39] Ichinohe T, Pang IK, Kumamoto Y, Peaper DR, Ho JH (2011). Microbiota regulates immune defense against respiratory tract influenza A virus infection.. Proc Natl Acad Sci U S A.

[40] Maslowski KM, Vieira AT, Ng A, Kranich J, Sierro F (2009). Regulation of inflammatory responses by gut microbiota and chemoattractant receptor GPR43.. Nature.

[41] Fukuda S, Toh H, Hase K, Oshima K, Nakanishi Y (2011). *Bifidobacteria* can protect from enteropathogenic infection through production of acetate.. Nature.

[42] Vintini E, Villena J, Alvarez S, Medina M (2010). Administration of a probiotic associated with nasal vaccination with inactivated *Lactococcus lactis*-PppA induces effective protection against pneumoccocal infection in young mice.. Clin Exp Immunol.

[43] Elinav E, Strowig T, Kau AL, Henao-Mejia J, Thaiss CA (2011). NLRP6 inflammasome regulates colonic microbial ecology and risk for colitis.. Cell.

[44] Kukkonen K, Nieminen T, Poussa T, Savilahti E, Kuitunen M (2006). Effect of probiotics on vaccine antibody responses in infancy–a randomized placebo-controlled double-blind trial.. Pediatr Allergy Immunol.

[45] Mullie C, Yazourh A, Thibault H, Odou MF, Singer E (2004). Increased poliovirus-specific intestinal antibody response coincides with promotion of *Bifidobacterium longum-infantis* and *Bifidobacterium breve* in infants: a randomized, double-blind, placebo-controlled trial.. Pediatr Res.

[46] Isolauri E, Joensuu J, Suomalainen H, Luomala M, Vesikari T (1995). Improved immunogenicity of oral D×RRV reassortant rotavirus vaccine by *Lactobacillus casei* GG.. Vaccine.

[47] Fang H, Elina T, Heikki A, Seppo S (2000). Modulation of humoral immune response through probiotic intake.. FEMS Immunol Med Microbiol.

[48] Soh SE, Ong DQ, Gerez I, Zhang X, Chollate P (2010). Effect of probiotic supplementation in the first 6 months of life on specific antibody responses to infant hepatitis B vaccination.. Vaccine.

[49] Remus DM, Kleerebezem M, Bron PA (2011). An intimate tête-à-tête - how probiotic *lactobacilli* communicate with the host.. Eur J Pharmacol.

[50] Rescigno M (2011). The intestinal epithelial barrier in the control of homeostasis and immunity.. Trends Immunol.

[51] Wells JM (2011). Immunomodulatory mechanisms of *lactobacilli*.. Microb Cell Fact.

[52] Marco ML, Pavan S, Kleerebezem M (2006). Towards understanding molecular modes of probiotic action.. Curr Opin Biotechnol.

